# Culturally Adapted School-Based Suicide Prevention Program for Pakistani Adolescents: Feasibility, Acceptability, and Preliminary Outcomes

**DOI:** 10.1192/bjo.2024.126

**Published:** 2024-08-01

**Authors:** Sehrish Irshad, Tayyeba Kiran, Nasim Chaudhry, Nusrat Husain, Maria Panagioti

**Affiliations:** 1 Pakistan Institute of Living and Learning (PILL), Karachi, Pakistan; 2 University of Manchester, Manchester, United Kingdom

## Abstract

**Aims:**

Suicide is one of the leading causes of death among young people. For adolescents, schools are considered as the best place to identify and respond to youth suicide risk but evidence on culturally sensitive, school-based suicide prevention programs is limited in low-resource settings such as Pakistan. This study aims to explore the perspectives of students, parents, teachers, and general practitioners regarding cultural adaptation, participation experiences, identified areas for improvement, and recommendations for scaling up the school-based suicide prevention program in Pakistan.

**Methods:**

This qualitative study was nested in a pilot randomized controlled trial that aimed at exploring the feasibility, acceptability and preliminary effectiveness of three suicide prevention interventions: (1) Linking Education and Awareness of Depression and Suicide-LEADS training for students (12–17 years), (2) Question-Persuade-Refer (QPR) training for teachers and parents and (3) Professional screening of at-risk students (ProfScreen) for health practitioners. A total of 8 focus group discussion (FGDs) were conducted at pre- and post-intervention stage with each type of stakeholder (students, teachers, parents and health professionals) by trained qualitative researchers using the semi-structured topic guides. Each FGDs involved a detailed presentation on intervention, sharing videos and educational posters.

**Results:**

As a result of pre-intervention FGDs, adaptations were made in the content of the interventions and were further refined through consultations with Patient and Public Involvement and Engagement group. All stakeholders who participated in post-intervention FGDs marked this suicide prevention program as feasible, acceptable and helpful in both identifying the risk of and preventing self-harm and suicide among young individuals, while also enhancing treatment pathways. Stakeholders perceived the interventions as valuable in augmenting knowledge about mental health, understanding the impact of mental health challenges on functioning, reducing stigma, and providing stakeholders with the necessary skills to identify and guide at-risk individuals. Teachers and parents endorsed the importance of discussing issues with children. Improvement in clinical practice of clinicians and teaching practice of teachers as well as understanding others’ behaviors were also reported.

**Conclusion:**

This study highlights potential role of culturally adapted school-based youth suicide prevention program for settings where rates of suicide are high and there are limited mental healthcare resources in addition to limited access to healthcare. School-based suicide prevention program is perceived as helpful in improving knowledge, attitudes, and help-seeking behaviours in adolescents.

